# Monitoring measurable residual disease and chimerism in patients with *JAK2* V617F-positive myelofibrosis after allogeneic hematopoietic cell transplantation

**DOI:** 10.1038/s41408-023-00867-x

**Published:** 2023-06-26

**Authors:** Jong-Mi Lee, Ari Ahn, Eun Jeong Min, Sung-Eun Lee, Myungshin Kim, Yonggoo Kim

**Affiliations:** 1 Department of Laboratory Medicine, Seoul St. Mary’s Hospital, College of Medicine, The Catholic University of Korea, Seoul, Republic of Korea; 2 Catholic Genetic Laboratory Center, College of Medicine, The Catholic University of Korea, Seoul, Republic of Korea; 3 Department of Laboratory Medicine, Incheon St. Mary’s Hospital, College of Medicine, The Catholic University of Korea, Seoul, Republic of Korea; 4 Department of Medical Life Sciences, College of Medicine, The Catholic University of Korea, Seoul, Republic of Korea; 5 Department of Hematology, Seoul St. Mary’s Hospital, College of Medicine, The Catholic University of Korea, Seoul, Republic of Korea; 6 Leukemia Research Institute, College of Medicine, The Catholic University of Korea, Seoul, Republic of Korea

Dear Editor,

Myelofibrosis (MF) is the most severe form of myeloproliferative neoplasm (MPN). Allogeneic hematopoietic stem cell transplantation (allo-HCT) is the only known curative option for MF. However, a significant proportion of allo-HCT recipients experience relapse. In 2013, the International Working Group (IWG)-MPN and European LeukemiaNet (ELN) suggested a definition for complete remission and relapse in MF [1]. Although this criterion is widely used, it does not consider chimerism and variable resolution of fibrosis in the post-allo-HCT setting. Given the usual complications of transplantation, reliance on conventional strategies may significantly delay effective post-transplantation interventions. In 2021, the European Blood and Marrow Transplantation (EBMT) group emphasized the role of molecular data in defining relapse after allo-HCT [2]; however, this still needs to be validated and supported by accumulated data. In this study, we measured measurable residual disease (MRD) and chimerism to define relapse and investigated their prognostic impact on MF after allo-HCT. We then sought to optimize the threshold and time points for predicting early relapse. This study was approved by the Institutional Review Board of Seoul St. Mary’s Hospital, Seoul, South Korea (KC22RISI0120).

We enrolled 34 patients with primary or secondary MF with mutated *JAK2* V617F who underwent allo-HCT at Seoul St. Mary’s Hospital between 2012 and 2021. All patients received a reduced-intensity conditioning regimen consisting of fludarabine (30 mg/m^2^ for 5 days) and busulfan (3.2 mg/kg for 2 days) with total body irradiation (TBI) of 200–400 cGy [3]. A total of 150 samples were obtained at the time of allo-HCT (*n* = 33) and on days +30 (*n* = 32), +100 (*n* = 31), +180 (*n* = 30), and +360 (*n* = 24) after allo-HCT. As the primary endpoint, overt relapse was determined using morphological and clinical criteria based on the EBMT definition [1, 2]. In addition to overt relapse, the prognostic relevance of cytogenetic changes (relapse or evolution), molecular relapse, and chimerism relapse was also investigated. The details of the relapse criteria and testing methods are described in the Supplementary Methods.

Six patients experienced overt relapse at a median of 7.5 months (range: 3.3–14.7 months) after allo-HCT. Eight patients died of overt relapse (*n* = 3), infection (*n* = 3), chronic graft-versus-host disease (*n* = 1), or other causes (*n* = 1). The median follow-up duration was 20.4 months after allo-HCT (95% confidence interval [CI]: 15.0–81.3 months). The 2-year overall survival (OS) was 72.1% (95% CI: 50.9–85.4%). The median relapse-free survival (RFS) was 18.9 months (95% CI: 14.0–42.3). The 1-year cumulative incidences of relapse (CIR) and unrelapse mortality were 14.7% (95% CI: 5.4–28.5%) and 11.8% (95% CI: 3.7–24.9%), respectively (Supplementary Table 1).

*JAK2*-MRD was measured using real-time PCR (JAK2 MutaQuant kit, Ipsogen, Qiagen). A total of 93.9% (31/33) of the patients had detectable *JAK2*-MRD (>0.014%) at the time of allo-HCT, with a median variant allele frequency (VAF) of 52.5% (95% CI: 32.9–71.7%) (Fig. 1A). Approximately half of the patients were positive *for JAK2*-MRD 1 year after allo-HCT: 62.5% on day +30, 48.4% on day +100, 46.7% on day +180, and 50% on day +360. Considering the long-lasting *JAK2*-MRD is a frequently observed phenomenon in MF, particularly in cases of reduced-intensity allo-HCT [4, 5], *JAK2*-MRD positivity at certain time points has limited prognostic significance. In terms of allele burden, *JAK2*-MRD VAF was higher in relapsed patients than in unrelapsed patients on days +100 and +180 (*P* = 0.005 and 0.011, respectively) (Fig. 1B). Receiver operating characteristic (ROC) analysis indicated that *JAK2*-MRD VAF on day +100 was a significant predictor of overt relapse (*P* < 0.001). The optimal *JAK2*-MRD VAF threshold was 0.021%, and the area under the ROC curve (AUC) was 0.877, with 100% sensitivity and 70% specificity (Fig. 1C, dotted line). In the analysis using the *JAK2*-MRD ratio (ratio of VAF at each time point to the previous VAF), the optimal threshold was ≥3-fold increase at day +100, and the AUC value increased up to 0.983 with 100% sensitivity and 91.3% specificity (Fig. 1C, solid line). Time-dependent ROC analysis also revealed that the *JAK2*-MRD ratio on day +100 showed the best performance, with an AUC value of 0.986–1.000 (Supplementary Table 2, Supplementary Fig. 1A, B). Compared with previous studies that reported the critical time point for *JAK2*-MRD as +180 days [4–6], the *JAK2*-MRD ratio (≥3-fold) on day +100 was an earlier indicator of overt relapse. Moreover, it is also feasible in routine schedules according to the EBMT guidelines, which recommend MRD monitoring at 30, 100, 180, 270, and 360 days after allo-HCT [2].

**Fig. 1:**
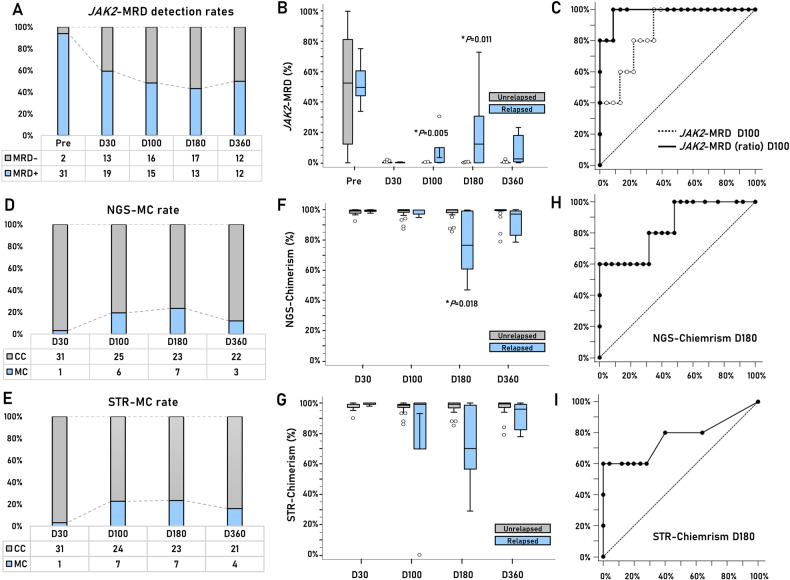
Investigation of best-performing molecular markers for overt relapse. **A** *JAK2*-MRD detection rate during 1 year of follow-up. **B** Comparison of *JAK2*-MRD VAF between relapsed and unrelapsed patients at different time points. **C** ROC curves of the *JAK2*-MRD VAF and *JAK2*-MRD ratio at D100. The *JAK2*-MRD VAF at D100 (dotted line) showed an AUC value of 0.877 and an optimal threshold of 0.021% with 100% sensitivity and 70% specificity (*P* < 0.001). The *JAK2*-MRD ratio at D100 (solid line) showed the best discriminative power for overt relapse (AUC: 0.983) at an optimal threshold of 2.877% with 100% sensitivity and 91.3% specificity (*P* < 0.001). **D** and **E** MC (donor chimerism <95%) rates by NGS and STR during 1 year of follow-up. **F** and **G** Comparison of donor chimerism measured by NGS and STR according to the over-relapse occurrences at different time points. Significant difference in the donor chimerism between the relapsed and unrelapsed patients was found in only NGS-chimerism at D180. **H** NGS-chimerism at D180 AUC value of 0.840 and optimal threshold of 76.63% with 60% sensitivity and 100% specificity (*P* = 0.001), but **I** STR-chimerism D180 showed no significant AUC values (*P* = 0.073). VAF variant allele frequency, ROC receiver operating characteristic, AUC area under the curve, MC mixed chimerism, CC complete chimerism, NGS next-generation sequencing, STR short tandem repeat.

In addition to MRD, chimerism monitoring is essential for assessing the degree of engraftment and the risk of relapse in MF [7, 8]. Donor chimerism was measured using next-generation sequencing (NGS; Devyser, Stockholm, Sweden) [9] and short tandem repeats (STR; Applied Biosystems, Warrington, UK) [10]. The mixed chimerism (MC, ≤95%) rates were 3.1% (3.1%), 22.6% (19.4%), 23.3% (23.3%), and 12.6% (16.0%) on days +30, +100, +180, and +360, respectively, by NGS and STR (data in parentheses) (Fig. 1D, E). As shown in Fig. 1F, G, a significant difference in donor chimerism between relapsed and unrelapsed patients was observed only in NGS-chimerism on day +180 (*P* = 0.018). ROC analysis also confirmed that NGS-chimerism on day +180 was a significant predictor of overt relapse (*P* = 0.001). The optimal NGS-chimerism threshold was 77%, with an AUC value of 0.840, leading to 100% sensitivity and 60% specificity (Fig. 1H, I). Time-dependent ROC analysis also revealed that NGS-chimerism at day +180 was the best predictor of overt relapse, with an AUC value of 0.834–0.932 (Supplementary Table 2, Supplementary Fig. 1C, D). Thus, our data indicated that high-level MC (≤77%) on day +180 was a significant marker for predicting overt relapse, with 100% specificity. Notably, a previous study comparing two conditioning regimens (two alkylating agents vs. one alkylating agent) found a significant association between MC on day +30 and relapse risk [11]. In our study, patients received one alkylating agent combined with TBI, resulting in little association between MC on day +30 and relapse, which may have been affected by various factors, including the conditioning regimen, TBI, and specimen types. However, further studies are required to explore this.

Figure 2A and Supplementary Fig. 2 depict the scenarios of 15 patients who showed emerging molecular markers of relapse. Overall, an increased *JAK2*-MRD ratio (≥3-fold), MC (≤95%), high-level MC (≤77%), and cytogenetic changes (relapse or evolution) were observed in 14, 10, 4, and 5 patients, respectively. The majority of cytogenetic changes involved cytogenetic evolution (80%, 4/5), whereas cytogenetic relapse was observed in only one patient (#23). These cytogenetic changes tend to occur during overt relapse. Therefore, our findings support the idea that cytogenetic changes in MPN are associated with disease progression [12].

**Fig. 2:**
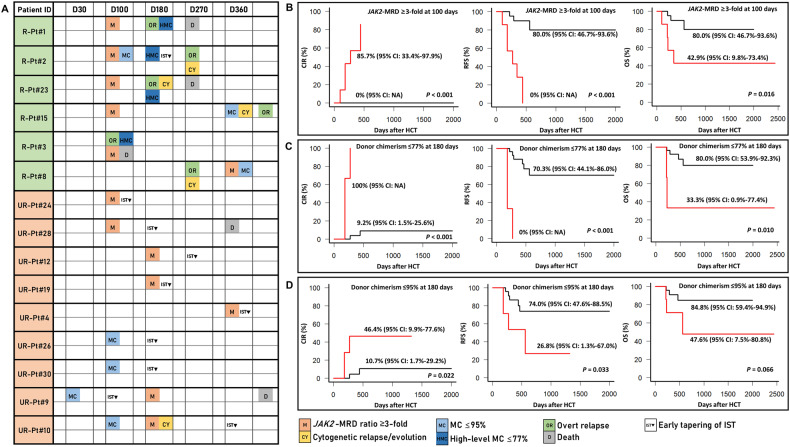
Chronology of emerging relapse evidence and survival graphs. **A** Swimmer plot of the 15 patients with relapse evidence and their outcomes, including 6 relapsed and 9 unrelapsed patients. The values of donor chimerism and *JAK2*-MRD VAF are presented in Supplementary Fig. 2. **B** Probability of overt relapse, relapse-free survival, and overall survival by *JAK2*-MRD ratio ≥3-fold at day +100. **C** Donor chimerism ≤77% at day +180, and **D** Donor chimerism ≤95% at day +180. Red positive, black negative. R-pt relapsed patient, UR-Pt unrelapsed patient, D day, MRD measurable residual disease, MC mixed chimerism, IST immunosuppressive therapy, VAF variant allele frequency, HCT hematopoietic stem cell transplantation, CI confidential interval, NA not available, MRD measurable residual disease, CIR cumulative incidence of relapse, RFS relapse-free survival, OS overall survival.

An increased *JAK2*-MRD ratio (≥3-fold) appeared first in five relapsed patients (83%, 5/6), 134 ± 130 days before overt relapse. One exceptional case (#8) presented with an overt relapse with cytogenetic evolution at day +270, an increased *JAK2*-MRD ratio, and MC (≤95%) caught up late on day +360. Monitoring molecular markers provides not only information for early relapse but also the depth of disease remission to guide therapeutic interventions [13, 14]. Nine patients who initially had positive molecular markers did not eventually progress to an overt relapse. Various combinations of markers were observed in these patients. Five patients showed only an increased *JAK2*-MRD ratio, two patients showed only MC, and the remaining two patients showed MC followed by an increased *JAK2*-MRD ratio with or without cytogenetic evolution. It is worth noting that early tapering of the immunosuppressive therapy (IST) was done in these patients, and the measured intermediate MC (77–95%) was frequently converted to full chimerism (>95%) after tapering. Meanwhile, one patient (#2) with high-level MC (≤77%) on day +180 eventually developed overt relapse despite discontinuation of IST. These results suggest that early tapering of IST upon persistence or emerging molecular markers might prevent overt relapse, possibly through the strong graft-versus-tumor effect of MF, and that the MC status provides a useful indicator for additional interventions [7, 15]. Our survival data (sTable 3) also revealed that an increased *JAK2*-MRD ratio (≥3-fold) at day +100 and high-level MC (≤77%) at day +180 was significantly associated with a CIR, RFS, and OS (Fig. 2B, C). MC (≤95%) on day +180 was significantly associated with CIR and RFS but not with OS (Fig. 2D).

In summary, *JAK2*-MRD was found to be a sensitive and early detector of relapse, but it frequently remained detectable for over a year. Therefore, serial assessments at short intervals would be beneficial, and an optimal threshold needs to be established. In this study, the *JAK2*-MRD ratio ≥3-fold at days +100 was related to relapse. Chimerism is a specific marker for relapse, especially 180 days after allo-HCT. Although we included a limited number of patients, our results supported the prognostic relevance of *JAK2*-MRD in chimerism.

## Supplementary information

Supplemental Material

## Data Availability

The datasets generated and/or analyzed during the current study are available from the corresponding author on reasonable request.
