# Supplementary material for: Monitoring measurable residual disease and chimerism in patients with JAK2 V617F-positive myelofibrosis after allogeneic hematopoietic cell transplantation
Source: Blood Cancer J. 2023 Jun 26;13(1):97. doi: 10.1038/s41408-023-00867-x (PMC10293273; doi:10.1038/s41408-023-00867-x)
Supplement: Supplementary file 1 — Supplemental Material [file 41408_2023_867_MOESM1_ESM.docx]

**Supplementary materials**

1. **Supplementary methods**
   1. **Definitions for relapse**

The overt relapse status was established based on the EBMT definition [1, 2]. The morphological and clinical criteria include the following: an increase in age-adjusted cellularity with an abnormal Myeloid:Erythroid ratio, typical megakaryocytic abnormalities, an increase in the grade of myelofibrosis, and/or development of myelodysplasia, monocytosis, or increased blast count, accompanied by irreversible cytopenia (hemoglobin <100 g/L, neutrophil count <1x10^9^/L, and platelet count <100 x10^9^/L) or an increased immature myeloid cell count in the peripheral blood. In addition to overt relapse according to the morphological and clinical criteria, the prognostic relevance of cytogenetic relapse or evolution, molecular relapse, and chimerism relapse was investigated. Cytogenetic relapse was defined as the appearance of a pre-existing cytogenetic abnormality, cytogenetic evolution, or new development of any abnormality confirmed by repeated testing. Molecular and chimerism relapses were assessed by *JAK2*-MRD and chimerism tests, respectively. The optimized thresholds and time points for molecular and chimerism relapses were also investigated.

- 1. **Transplantation procedures**

All patients received a reduced-intensity conditioning regimen consisting of fludarabine (30 mg/m^2^ for 5 days) and busulfan (3.2 mg/kg for 2 days) with total body irradiation (TBI) of 200–400 cGy [3]. Graft-Versus-Host disease (GVHD) prophylaxis consisted of antithymocyte globulin (ATG; Thymoglobulin^®^), a calcineurin inhibitor, and methotrexate (MTX). ATG was administered at a dose of 2.5–7.5 mg/kg according to the donor types (≥5 mg/kg in mismatched donor transplantation). MTX (5 mg/m^2^) was administered on days +1, +3, +6, and +11, along with calcineurin inhibitors (cyclosporine for matched-sibling donors and tacrolimus for unrelated donors and haploidentical familial donors). The calcineurin inhibitor dose was tapered gradually starting on days 100–120 after allo-SCT without acute GVHD. Early tapering of immunosuppressive therapy (IST) was performed in patients clinically suspected of relapse. The other general transplantation procedures were performed as described previously [4, 5].

- 1. **MRD monitoring using *JAK2* V617F quantification**

DNA was extracted from the bone marrow or peripheral blood using QIAsymphony DSP DNA kits with a QIAsymphony instrument (Qiagen, Hilden, Germany). Quantification was performed using the Qubit dsDNA Broad Range Assay kit (Thermo Fisher Scientific, Waltham, MD, USA). MRD monitoring for *JAK2* V617F was performed using real-time PCR (JAK2 MutaQuant kit, Ipsogen, Qiagen) according to the manufacturer’s instructions. Briefly, a short amplicon covering the *JAK2* V617F region was amplified using 25 ng of purified DNA. Positive and negative calibrators at four different concentrations were included in each run to obtain standard curves. All samples were tested in duplicate, and the mean cycle threshold (Ct) values were transformed to copy numbers of *JAK2* V617F and wild-type using the prepared standard curves. *JAK2*-MRD was expressed as the variant allele frequency (VAF) as a percentage of *JAK2* V617F copy number for total *JAK2* (*JAK2* V617F plus *JAK2* wild-type) copy number. Based on the assay’s sensitivity of 0.014 %, we set the threshold for J*AK2*-MRD positivity at VAF > 0.014%.

We developed the other *JAK2*-MRD marker, which represents the change in *JAK2* V617F, by calculating the ratio of the VAF at each time point to the previous VAF. The *JAK2*-MRD ratio was calculated at three different time points using the following method:

JAK2-MRD ratio on day +100 = (JAK2-MRD VAF (%) on day +100) / (JAK2-MRD VAF (%) on day +30)

JAK2-MRD ratio on day +180 = (JAK2-MRD VAF (%) on day +180) / (JAK2-MRD VAF (%) on day +100)

JAK2-MRD ratio on day +360 = (JAK2-MRD VAF (%) on day +360) / (JAK2-MRD VAF (%) on day +180)

- 1. **Chimerism monitoring**

We monitored the percentage of donor chimerism using both a next-generation sequencing-based assay (NGS chimerism) and a short tandem repeat-based assay (STR chimerism). NGS chimerism was analyzed using Devyser chimerism NGS kits (Devyser, Stockholm, Sweden). In a single tube, 24 insertion–deletion mutation markers were sequenced in a single tube using MiSeq (Illumina, San Diego, CA, USA). The data were analyzed using a dedicated program.

STR chimerism was assessed using AmpFlSTR Identifier PCR Amplification (Applied Biosystems, Warrington, UK), as previously reported [6, 7]. Briefly, 16 STR markers were amplified. PCR was performed using a C1000 Touch Thermal Cycler (Bio-Rad Laboratories Inc., Hercules, CA, USA). Amplified PCR products were analyzed via capillary electrophoresis using an ABI 3130xl genetic analyzer (Applied Biosystems, Foster City, CA, USA). GeneMapper ID Software Version 4.1 (Applied Biosystems, Foster City, CA, USA) was used for automated genotyping and quantification of the peak areas.

- 1. **Statistics**

The endpoints were overt relapse and death. Patient characteristics were expressed as medians and ranges for continuous variables and frequencies for categorical variables. Categorical data were compared using the Fisher’s exact test or the χ^2^ test, and continuous data were compared using the Wilcoxon test. We analyzed the predictive power of the clinical and molecular factors for overt relapse, non-relapse mortality (NRM), relapse-free survival (RFS), and overall survival (OS). We defined RFS as the time from allo-HCT to relapse or death and OS as the time from allo-HCT to death from any cause. Medical records were tracked until October 2022. Receiver operating characteristic (ROC) curve analysis was performed to determine the optimal threshold and time points for *JAK2*-MRD and chimerism to predict overt relapse.

The thresholds and time points obtained were validated via time-dependent ROC analysis in the competing risk setting using the time ROC package in R [8]. Relapse and NRM were considered competing risk events. The area under the curve (AUC) was computed on days +300 and +500 after allo-HCT. RFS and OS were estimated using the Kaplan–Meier method. Competing risk analysis was performed to estimate the probability of the cumulative incidence of relapse (CIR) and NRM. The CIR was compared across groups using the Gray test and cmprsk module in R [9, 10]. RFS and OS were compared using the Cox proportional hazards regression analysis. Continuous values of *JAK2*-MRD and chimerism at different time points were evaluated as time-dependent covariates. Statistical analyses were performed using MedCalc version 19.1.7 (MedCalc Software, Ostend, Belgium) and R software version 4.1.3 (R Foundation for Statistical Computing, Vienna, Austria).

**References**

1. Tefferi, A, Cervantes, F, Mesa, R, Passamonti, F, Verstovsek, S, Vannucchi, AM, et al. Revised response criteria for myelofibrosis: International Working Group-Myeloproliferative Neoplasms Research and Treatment (IWG-MRT) and European LeukemiaNet (ELN) consensus report. Blood. 2013; 122:1395-1398.

2. McLornan, DP, Hernandez-Boluda, JC, Czerw, T, Cross, N, Joachim Deeg, H, Ditschkowski, M, et al. Allogeneic haematopoietic cell transplantation for myelofibrosis: proposed definitions and management strategies for graft failure, poor graft function and relapse: best practice recommendations of the EBMT Chronic Malignancies Working Party. Leukemia. 2021; 35:2445-2459.

3. Kim DH, Seo J, Shin D-Y, Koh Y, Hong J, Kim I, et al. Reduced-intensity conditioning versus myeloablative conditioning allogeneic stem cell transplantation for patients with myelofibrosis. Blood Res. 2022;57:264-271.

4. Lee, S-E, Lim, J-Y, Kim, TW, Jeon, Y-W, Yoon, J-H, Cho, B-S, et al. Matrix metalloproteinase-9 in monocytic myeloid-derived suppressor cells correlates with early infections and clinical outcomes in allogeneic hematopoietic stem cell transplantation. Biol Blood Marrow Transplant. 2018; 24:32-42.

5. Lee, S-E, Lim, J-Y, Ryu, D-B, Kim, TW, Park, SS, Jeon, Y-W, et al. Alteration of the intestinal microbiota by broad-spectrum antibiotic use correlates with the occurrence of intestinal graft-versus-host disease. Biology of Blood and Marrow Transplantation. 2019; 25:1933-1943.

6. Lee, J-M, Kim, Y-J, Park, S-S, Han, E, Kim, M, Kim, Y. Simultaneous monitoring of mutation and chimerism using next-generation sequencing in myelodysplastic syndrome. J Clin Med. 2019; 8:2077.

7. Han, E, Kim, M, Kim, Y, Han, K, Lim, J, Kang, D, et al. Practical informativeness of short tandem repeat loci for chimerism analysis in hematopoietic stem cell transplantation. Clin Chim Acta. 2017; 468:51-59.

8. Blanche, P, Dartigues, JF, Jacqmin‐Gadda, H. Estimating and comparing time‐dependent areas under receiver operating characteristic curves for censored event times with competing risks. Stat Med. 2013; 32:5381-5397.

9. Scrucca, L, Santucci, A, Aversa, F. Regression modeling of competing risk using R: an in depth guide for clinicians. Bone Marrow Transplant. 2010; 45:1388-1395.

10. Scrucca, L, Santucci, A, Aversa, F. Competing risk analysis using R: an easy guide for clinicians. Bone Marrow Transplant. 2007; 40:381-387.

1. **Supplementary tables**

**sTable 1. Patient demographics**

| Patients and disease characteristics | | Total (n=34) | Unrelapsed (n=28) | Relapsed (n=6) | *P* value |
| --- | --- | --- | --- | --- | --- |
| Age | Year, median (range) | 62.5 (57-67) | 63.0 (58.4-66.6) | 59.0 (50.2-66.8) | 0.319 |
|  | <65 years | 21 (61.8%) | 17 (60.7%) | 4 (66.7%) | 0.300 |
|  | ≥65 years | 13 (38.2%) | 11 (39.3%) | 2 (33.3%) |  |
| Sex | Male | 22 (64.7%) | 17 (60.7%) | 4 (66.7%) | 0.300 |
|  | Female | 12 (35.3%) | 11 (39.3%) | 2 (33.3%) |  |
| Diagnosis | PMF | 23 (67.6%) | 21 (75.0%) | 2 (33.3%) | 0.141 |
|  | Post PV/ET MF | 11 (32.4%) | 7 (25.0%) | 4 (66.7%) |  |
| BM blast at transplantation | <5% | 29 (85.3%) | 23 (82.1%) | 0 (0.0%) | 0.739 |
|  | ≥10% | 5 (14.7%) | 5 (17.9%) | 0 (0.0%) |  |
| IPSS at diagnosis | Low to intermediate-1 | 3 (8.8%) | 2 (7.1%) | 1 (16.7%) | 0.485 |
|  | Intermediate-2 | 13 (38.2%) | 10 (35.7%) | 3 (50.0%) |  |
|  | High | 18 (52.9%) | 16 (57.1%) | 2 (33.3%) |  |
| DIPSS at transplant | Intermediate -1 | 4 (11.8%) | 3 (10.7%) | 1 (16.7%) | 0.191 |
|  | Intermediate -2 | 18 (52.9%) | 15 (53.6%) | 3 (50.0%) |  |
|  | High | 12 (35.3%) | 10 (35.7%) | 2 (33.3%) |  |
| DIPSS Plus at transplant | Intermediate -1 | 2 (5.9%) | 1 (3.6%) | 1 (16.7%) | 0.349 |
|  | Intermediate -2 | 18 (52.9%) | 16 (57.1%) | 2 (33.3%) |  |
|  | High | 14 (41.2%) | 11 (39.3%) | 3 (50.0%) |  |
| ABO incompatibility | Matched | 20 (58.8%) | 17 (60.7%) | 3 (50.0%) | 0.634 |
|  | Mismatched | 14 (41.2%) | 11 (39.3%) | 3 (50.0%) |  |
| Donor type | Matched sibling | 17 (50.0%) | 15 (53.6%) | 2 (33.3%) | 0.523 |
|  | Matched unrelated | 10 (29.4%) | 7 (25.0%) | 3 (50.0%) |  |
|  | Mismatched unrelated | 4 (11.8%) | 3 (10.7%) | 1 (16.7%) |  |
|  | Haploidentical | 3 (8.8%) | 3 (10.7%) | 0 (0.0%) |  |
| Transplant outcomes | | **Total (n=34)** | **Unrelapsed (n=28)** | **Relapsed (n=6)** | ***P* value** |
| Median follow-up duration for survivors (95% CI) | | 613 (451-2,440) | 566 (420-2,001) | 827 (636-2,440) | 0.176 |
| Death | | 8 (23.5%) | 5 (17.9%) | 3 (50%) | 0.097 |
| Median days to engraftment | ANC >0.5× 10^9^/L (95% CI) | 13.0 (13.0-14.2) | 13.0 (12.4-14.0) | 14.5 (10.6-22.3) | 0.397 |
|  | Platelets >20× 10^9^/L (95% CI) | 16.0 (13.6-20.4) | 16.0 (14.0-19.9) | 18.5 (8.0-49.0) | 0.980 |
| Acute GVHD | (grade1-4) | 12 (35.3%) | 11 (39.3%) | 1 (16.7%) | 0.300 |
|  | (grade2-4) | 7 (20.6%) | 7 (25.0%) | 0 (0%) | 0.176 |
| Chronic GVHD | (mild-severe) | 16 (47.1%) | 14 (50.0%) | 2 (33.3%) | 0.465 |
|  | (moderate-severe) | 9 (26.5%) | 8 (28.6%) | 1 (16.7%) | 0.555 |

Abbreviations: CI, confidence interval; PMF, primary myelofibrosis; PV, polycythemia vera; ET, essential thrombocythemia; MF, myelofibrosis; BM, bone marrow; IPSS, International Prognostic Scoring System; DIPSS, Dynamic International Prognostic Scoring System; GVHD, graft-versus-host disease.

**sTable 2. AUC values calculated by time-dependent ROC analysis**

| Variable | Measured time | AUC at D300 | AUC at D500 |
| --- | --- | --- | --- |
| *JAK2*-MRD VAF | D30 | 0.404 | 0.309 |
|  | D100 | 0.918 | 0.787 |
|  | D180 | 0.955 | 0.750 |
|  | D360 | - | 0.753 |
| *JAK2*-MRD ratio | D100 | 1.000 | 0.986 |
|  | D180 | 0.743 | 0.565 |
|  | D360 | - | 0.680 |
| NGS chimerism | D30 | 0.447 | 0.442 |
|  | D100 | 0.692 | 0.583 |
|  | D180 | 0.932 | 0.834 |
|  | D360 | - | 0.655 |
| STR chimerism | D30 | 0.301 | 0.429 |
|  | D100 | 0.508 | 0.439 |
|  | D180 | 0.926 | 0.728 |
|  | D360 | - | 0.691 |

Abbreviations: AUC, Area Under the ROC Curve; ROC, Receiver Operating Characteristic; MRD, measurable residual disease; VAF, Variant allele frequency; NGS, Next-generation sequencing; STR, Short Tandem Repeat; D, day.

**sTable 3. Survival analysis**

| **Univariate variables** | **Pt #** | **Cumulative incidence of overt relapse** | | **Cumulative incidence of NRM** | | **Relapse free survival** | | **Overall survival** | |
| --- | --- | --- | --- | --- | --- | --- | --- | --- | --- |
|  |  | **HR (95% CI)** | **P value** | **HR (95% CI)** | **P value** | **HR (95% CI)** | **P value** | **HR (95% CI)** | **P value** |
| ***JAK2*-MRD ratio at 100 days**  **<3-fold**  **≥ 3-fold** | 28  21  7 | **1**  **NA** | **<0.001** | 1  2.76 (0.34-22.7) | 0.345 | **1**  **15.91 (3.12-81.30)** | **<0.001** | **1**  **5.24 (1.17-23.48)** | **0.031** |
| **Donor chimerism at 180 days**  **>95%**  **≤ 95%** | 30  23  7 | **1**  **6.41 (1.20-34.11)** | **0.029** | 1  1.79 (0.30-10.62) | 0.522 | **1**  **3.89 (1.04-14.58)** | **0.044** | 1  4.02 (0.81-20.03) | 0.089 |
| **Donor chimerism at 180 days**  **>77%**  **≤ 77%** | 30  27  3 | **1**  **46.82 (7.23-303.25)** | **<0.001** | 1  NA | NA | **1**  **31.92 (4.99-204.36)** | **<0.001** | **1**  **6.98 (1.26-38.68)** | **0.026** |
| **Age group**  **< 65 years**  **≥ 65 years** | 34  21  13 | 1  0.808 (0.16-4.07) | 0.796 | 1  2.81 (0.54-14.60) | 0.219 | 1  1.41 (0.42-4.68) | 0.578 | 1  1.96 (0.81-4.73) | 0.133 |
| **Diagnosis**  **PMF**  **Secondary MF** | 34  23  11 | 1  4.37 (0.84-22.91) | 0.081 | 1  1.81 (0.34-9.79) | 0.489 | 1  1.87 (0.49-7.08) | 0.358 | 1  1.23 (0.50-3.01) | 0.658 |
| **BM blast at transplantation**  **< 5%**  **≥10%** | 34  29  5 | 1  NA | NA | **1**  **6.66 (1.19-37.11)** | **0.030** | 1  2.99 (0.91-9.89) | 0.072 | 1  0.60 (0.14-2.60) | 0.038  0.050 |
| **IPSS**  **Low to int-1**  **Int-2**  **High** | 34  3  13  18 | 1  0.77 (0.12-5.41)  0.32 (0.04-2.85) | 0.509  0.797  0.306 | 1  0.10 (0.22-4.57)  NA | NA  0.997  NA | 1  0.79 (0.08-7.58)  1.01 (0.12-8.27) | 0.934  0.836  0.990 | 1  NA  NA | 0.083  0.998  0.998 |
| **DIPSS**  **Int-1**  **Int-2**  **High** | 34  4  18  12 | 1  0.71 (0.10-5.21)  0.59 (0.06-5.65) | 0.900  0.740  0.646 | 1  NA | NA | 1  1.19 (0.14-10.19)  1.32 (0.15-11.43) | 0.966  0.876  0.804 | 1  NA  NA | 0.994  0.998  0.998 |
| **DIPSS Plus**  **Int-1**  **Int-2**  **High** | 34  2  18  14 | 1  0.21 (0.03-1.58)  0.36 (0.06-2.03) | 0.289  0.130  0.247 | 1  NA | NA | 1  0.31 (0.03-2.91)  0.56 (0.06-4.83) | 0.502  0.307  0.595 | 1  NA  NA | 0.995  0.998  0.998 |
| **ABO incompatibility**  **Matched**  **Mismatched** | 34  20  14 | 1  1.84 (0.43-7.89) | 0.410 | 1  1.61 (0.29-8.83) | 0.583 | 1  1.74 (0.52-5.79) | 0.366 | 1  3.85 (0.88-16.79) | 0.073 |
| **Donor type**  **Matched sibling**  **Matched unrelated**  **Mismatched unrelated**  **Haploidentical** | 34  17  10  4  3 | 1  3.20 (0.59-17.41)  1.94 (0.24-15.92)  NA | NA  0.179  0.537  NA | 1  1.05 (0.09-10.24)  NA  0.17 (0.92-36.56) | NA  0.971  NA  0.062 | 1  2.12 (0.53-8.51)  0.91 (1.10-8.14)  2.89 (0.53-15.87) | 0.530  0.287  0.931  0.222 | 1  4.49 (0.82-24.59)  NA  6.36 (0.89-45.46) | 0.255  0.084  0.999  0.066 |
| **Acute GVHD**  **No**  **Yes** | 34  22  12 | 1  0.31 (0.04-2.27) | 0.253 | 1  0.33 (0.04-2.72) | 0.302 | 1  0.31 (0.07-1.44) | 0.136 | 1  0.22 (0.03-1.79) | 0.157 |
| **Chronic GVHD**  **No**  **Yes** | 34  18  16 | 1  0.46 (0.10-2.15) | 0.321 | 1  0.18 (0.02-1.79) | 0.142 | 1  0.30 (0.08-1.15) | 0.078 | 1  0.12 (0.01-0.96) | 0.045 |

Abbreviations: Pt #, number of patients; HR, hazard ratio; NRM, NRM, Non-relapse mortality;, MRD, measurable residual disease; PMF, primary myelofibrosis; MF, myelofibrosis; BM, bone marrow; IPSS, International Prognostic Scoring System; DIPSS, Dynamic International Prognostic Scoring System; GVHD, graft-versus-host disease.

1. **Supplementary figures**


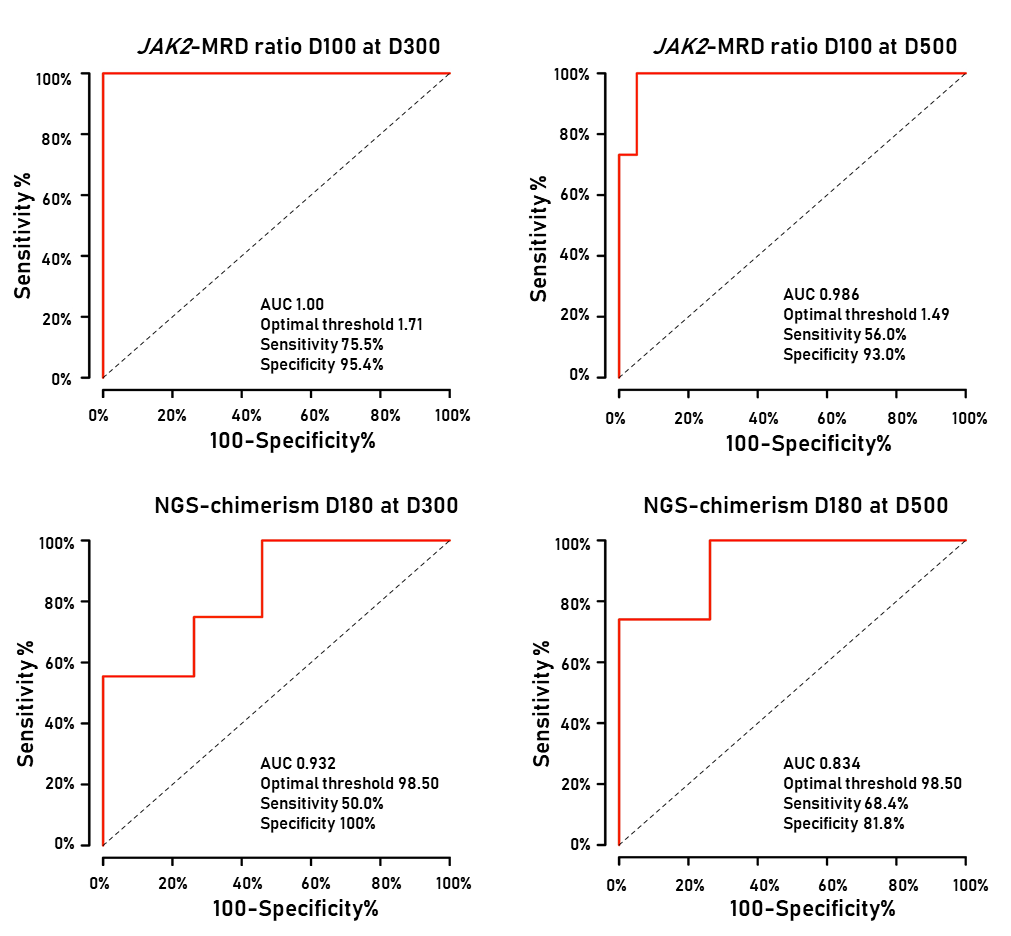


**C**

**D**

**B**

**A**

**sFigure 1. Time-dependent ROC curves of the overt relapse prediction markers.** (A) *JAK2*-MRD ratio at D100 for overt relapse prediction at D300 and (B) D500. (C) NGS chimerism at D180 for overt relapse prediction at D300 and (D) D500.

Abbreviations: AUC, Area Under the ROC Curve; ROC, Receiver Operating Characteristic; MRD, measurable residual disease; D, day; NGS, Next-generation sequencing.


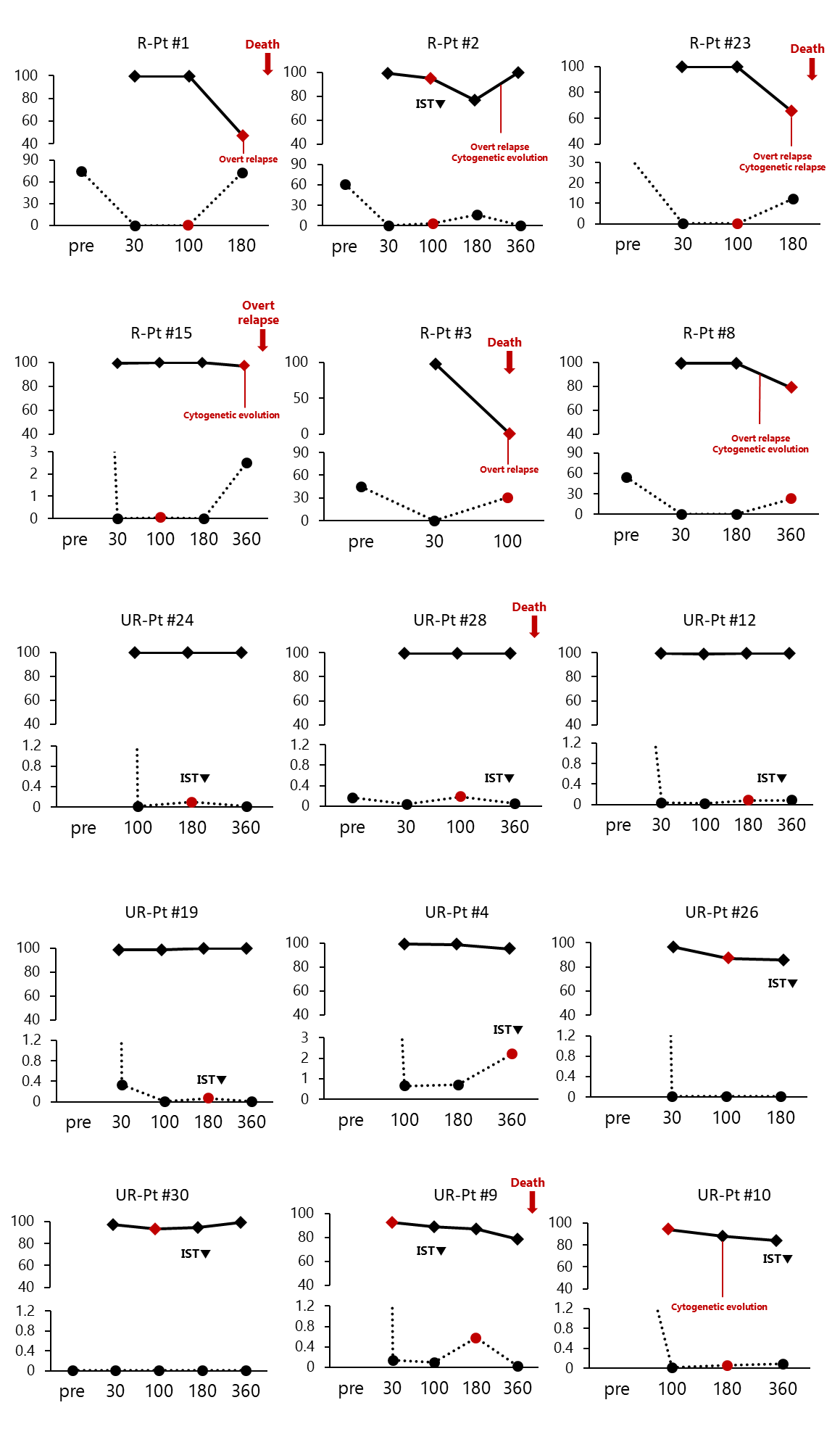


**sFigure 2. Dynamics of *JAK2*-MRD and chimerism.** The graphs show the molecular follow-up of 15 patients with emerging evidence of relapse; increased *JAK2*-MRD ratio (≥ 3-fold) and/or mixed chimerism (≤95%) marked by red dots. Solid lines and dotted lines display the quantification of donor chimerism (%) and *JAK2*-MRD VAF (%), respectively.

Abbreviations: R-Pt, relapsed patient; UR-Pt, unrelapsed patient; ITS▼, early tapering of immunosuppresive therapy; MRD, measurable residual disease; VAF, variant allele frequency.
